# Supplementary material for: Reusability Report: Evaluating the performance of a meta-learning foundation model on predicting the antibacterial activity of natural products
Source: Nat Mach Intell. 2026 Feb 12;8(2):270–5. doi: 10.1038/s42256-026-01187-y (PMC12932103; doi:10.1038/s42256-026-01187-y)
Supplement: Supplementary file 1 — Supplementary Methods, Tables 1–4 and Figs. 1–9. [file 42256_2026_1187_MOESM1_ESM.pdf]

nature machine intelligence

Supplementary information https://doi.org/10.1038/s42256-026-01187-y

Reusability Report: Evaluating the performance of a meta-learning foundation model on predicting the antibacterial activity of natural products

In the format provided by the authors and unedited

Supplementary Information

Table of Contents

1 Supplementary Methods 2

2 Supplementary Tables 5

3 Supplementary Figures 7

1 Supplementary Methods

Comparisons between the training and fine-tuning data:

We analyzed the differences between the ChEMBL training set, the BindingDB training set, and the antibacterial NPs fine-tuning dataset by comparing the distribution of the molecular properties of the compounds in each dataset (**Supplementary Table 1, Supplementary Figure 2**). RDKit[1] was used to compute the following descriptors: molecular weight (MW), the log of the partition coefficient (LogP), topological polar surface area (TPSA), number of hydrogen bond donors (HBD), and the number of hydrogen bond acceptors (HBA). We also compared the structural similarities of the compounds by computing the Fréchet ChemNet Distance (FCD), Maximum Mean Discrepancy (MMD), and scaffold novelty (ScaffNov) between the compounds in the fine-tuning and training datasets (**Supplementary Table 2**).

The FCD and MMD are distance metrics for determining the similarities between two distributions of molecules. We calculated the FCD by using the implementation provided by Preuer et al.[2] in their GitHub repository (https://github.com/bioinf-jku/FCD). We computed the MMD by following the implementation provided by Klarner et al.[3] (https://github.com/leojklarner/Q-SAVI). For both the FCD and MMD, we randomly selected 10,000 molecules from the ChEMBL and BindingDB training sets and calculated the distance metrics between these subsets and the entire antibacterial NPs dataset. The larger the FCD and MMD values, the larger the difference between the two distributions of molecules. In this study, we modified the definition of scaffold novelty defined by Grisoni et al.[4]. We calculated scaffold novelty by determining the number of Bemis-Murko scaffolds[5] in the fine-tuning dataset that are not present in the training set.

Additionally, we computed the scaled Shannon entropy (SSE) as defined by Medina-Franco et al.[6]. SSE is a measure of scaffold diversity, and a SSE of 0 indicates the presence of a single scaffold. The larger the SSE, the more scaffold diversity is present in the distribution. We calculated the SSE on the top 10, top 50, and top 100 most populated scaffolds in the fine-tuning and training datasets (**Supplementary Table 3**).

Analyzing assay overlap between the fine-tuning and training datasets

To determine if the bacterial assays in the antibacterial NPs dataset were present in the ChEMBL training set, we searched the ChEMBL database in two ways. First by searching ChEMBL for the references in the antibacterial NPs database. Second, by searching ChEMBL for growth inhibition assays containing the compounds in the antibacterial NPs datasets.

The antibacterial NPs dataset is a result of a manual literature review and contains the references from the original papers that the NPs were curated from. Using the ChEMBL webresource client[7], we used the DOIs of the references to determine if any of the documents had been deposited into ChEBML. We found nine references that had information deposited into ChEMBL. We removed the compounds associated with these nine references (35 total compounds) and fine-tuned ActFound, ActFound Transfer, MAML, ProtoNet, and TransferQSAR. Each model was fine-tuned using 20%, 40%, 60%, and 80% of the assay data for fine-tuning. Each assay was randomly split

40 times into the fine-tuning and testing sets, and the models were fine-tuned with each random split. The results of the models are an average across each iteration.

To search for identical growth inhibitory assays, we used the ChEMBL webresource client to search ChEMBL for assays of the same molecule/organism pairs that are in the antibacterial NPs dataset. We considered the assay a match and removed it from the NPs dataset if it had the same molecule/organism pair, the bao_label was equal to 'organism-based format', the standard type was equal to 'MIC', the standard units equal to 'ug/mL' and the standard relation equal to '='. Using these criteria, we identified and removed 324 molecule/organism pairs in the NPs dataset. We fine-tuned each model using the same procedure as when we fine-tuned after removing identical references (**Supplementary Figure 3,7-8**).

In addition to analyzing assay overlap, we also determined identical compounds between the antibacterial NPs datasets and the training datasets. To do this, we used RDKit to compute the Tanimoto similarity between the compounds in the NPs dataset and the compounds in the training datasets. We then removed compounds with a Tanimoto similarity score >0.8, >0.6, and >0.4 from the antibacterial NPs dataset and fine-tuned ActFound in the 16-shot setting. Each assay was randomly split 40 times into the fine-tuning and testing sets, and the model was fine-tuned with each random split. The results of the model are an average across each iteration. (**Supplementary Figure 1**).

Fine-tuning with a scaffold split

In addition to a random split, we also looked at fine-tuning ActFound on a scaffold split. In this setting, the molecules within each assay were split so that the compounds within the fine-tuning set were dissimilar to those in the testing set. We followed the 'realistic' split procedure established by Martin et al. which uses hierarchical clustering, a Tanimoto similarity-based clustering method[8]. We modified their implementation so that the clusters were randomized and then assigned to the fine-tuning and testing sets. Each assay was split 40 times into the fine-tuning and testing sets, and each model was fine-tuned with each scaffold split. We used 20%, 40%, 60%, and 80% of the assay data for fine-tuning. The results of the models are an average across each iteration (**Supplementary Figure 6,9**).

References:

1. RDKit: Open-source cheminformatics. https://www.rdkit.org
2. Preuer, K., Renz, P., Unterthiner, T., Hochreiter, S. & Klambauer, G. Fréchet ChemNet Distance: A Metric for Generative Models for Molecules in Drug Discovery. *J. Chem. Inf. Model.* **58**, 1736–1741 (2018).
3. Klarner, L. *et al.* Drug Discovery under Covariate Shift with Domain-Informed Prior Distributions over Functions. in *Proceedings of the 40th International Conference on Machine Learning* 17176–17197 (PMLR, 2023).
4. Grisoni, F., Moret, M., Lingwood, R. & Schneider, G. Bidirectional Molecule Generation with Recurrent Neural Networks. *J. Chem. Inf. Model.* **60**, 1175–1183 (2020).
5. Bemis, G. W. & Murcko, M. A. The Properties of Known Drugs. 1. Molecular Frameworks. *J. Med. Chem.* **39**, 2887–2893 (1996).

6. Medina-Franco, J. L., Martínez-Mayorga, K., Bender, A. & Scior, T. Scaffold Diversity Analysis of Compound Data Sets Using an Entropy-Based Measure. *QSAR & Combinatorial Science* **28**, 1551–1560 (2009).
7. Davies, M. *et al.* ChEMBL web services: streamlining access to drug discovery data and utilities. *Nucleic Acids Res* **43**, W612–W620 (2015).
8. Martin, E. J. *et al.* All-Assay-Max2 pQSAR: Activity Predictions as Accurate as Four-Concentration IC50s for 8558 Novartis Assays. *J. Chem. Inf. Model.* **59**, 4450–4459 (2019).

2 Supplementary Tables

Supplementary Table 1. Properties distributions of the antibacterial NPs fine-tuning dataset and the training datasets

	Mean ± SD	Median	Q1	Q3	IQR	Min	Max
**MW**							
NPs	452.3 ± 182.5 **a**, **b**	437.4	372.5	504.9	172.3	98.10	700.9
ChEMBL	462.5 ± 271.3	424.4	355.7	501.4	145.7	137.2	719.9
BindingDB	371.2 ± 221.3	340.4	270.2	442.5	132.4	174.0	703.4
**LogP**							
NPs	3.767 ± 1.836	3.791	2.788	4.813	2.972	-2.175	9.712
ChEMBL	3.703 ± 2.264	3.824	2.751	4.897	2.145	-0.467	8.115
BindingDB	3.730 ± 2.668	3.707	2.282	5.254	2.025	-0.249	7.851
**TPSA**							
NPs	93.55 ± 72.33	87.15	65.72	109.9	67.04	0	208.1
ChEMBL	97.54 ± 112.5	81.24	58.95	106.7	47.71	0	178.2
BindingDB	87.01 ± 88.96	74.60	40.46	107.5	44.14	0	176.1
**HBD**							
NPs	1.870 ± 2.613 **b**	2	1	2	2	0	6
ChEMBL	2.114 ± 4.047	1	1	2	1	0	3.5
BindingDB	2.510 ± 3.451	2	1	3	1	0	3.5
**HBA**							
NPs	5.889 ± 3.006 **a**, **b**	6	4	7	4	0	12
ChEMBL	5.886 ± 4.113	5	4	7	3	0	11.5
BindingDB	5.083 ± 4.407	5	2	6	3	0	11.5
**p(X)**							
NPs	-1.313 ± 0.526 **a**, **b**	-1.494	-1.796	-0.903	0.893	-2	0.409
ChEMBL	-2.236 ± 1.642	-2.252	-3.497	-1.041	2.455	-7.180	2.642
BindingDB	-2.112 ± 1.455	-2.021	-3.079	-1.079	2	-6.079	1.921

(MW = molecular weight, LogP = log10(partition coefficient), TPSA = topological polar surface area, HBD = number of hydrogen bond donors, HBA = number of hydrogen bond acceptors, p(X) = $-\log_{10}(X)$)

[a]Significant difference detected when compared to the ChEMBL training set when using a Tukey HSD post hoc test ($p < 0.05$)

[b]Significant difference detected when compared to the BindingDB training set when using a Tukey HSD post hoc test ($p < 0.05$)

Supplementary Table 2. Structural similarities between the antibacterial NPs fine-tuning dataset to the training datasets

Dataset	FCD	MMD	ScaffNov (%)
NPs to ChEMBL	43.9	0.208	61.4
NPs to BindingDB	49.1	0.224	70.7

(FCD = Frechet ChemNet Distance, MMD = Maximum Mean Discrepancy, ScaffNov = Scaffold Novelty)

Supplementary Table 3. Scaled Shannon Entropy (SSE) of the top 10, 50, and 100 most populated scaffolds in the fine-tuning and training datasets

**Dataset**	**SSE top 10**	**SSE top 50**	**SSE top 100**
NPs	0.966	0.927	0.924
ChEMBL	0.798	0.865	0.876
BindingDB	0.764	0.879	0.907

Supplementary Table 4. Bounds of the box plots in Supplementary Figure 2

	**Mean ± SD**	**Median**	**Q1**	**Q3**	**IQR**	**Min**	**Max**
**MW**							
NPs	358.2 ± 139.0	340.4	270.2	441.2	170.9	126.2	697.5
ChEMBL	443.7 ± 139.7	424.4	357.4	499.4	141.9	206.2	712.4
BindingDB	443.3 ± 102.4	437.4	374.4	503.5	129.1	214.1	697.1
**LogP**							
NPs	3.754 ± 2.134	3.707	2.288	5.214	2.926	-1.714	9.459
ChEMBL	3.774 ± 1.725	3.824	2.777	4.873	2.095	-0.366	8.016
BindingDB	3.797 ± 1.520	3.791	2.814	4.789	1.975	-0.149	7.752
**TPSA**							
NPs	89.50 ± 33.85	74.60	40.46	107.2	66.76	0	207.4
ChEMBL	89.04 ± 50.50	81.23	59.30	105.9	46.60	15.60	175.8
BindingDB	80.57 ± 50.92	87.14	66.32	109.9	42.96	19.03	173.7
**HBD**							
NPs	2.300 ± 2.073	2	1	3	2	0	6
ChEMBL	1.784 ± 1.822	1	1	2	1	0	3.5
BindingDB	1.695 ± 1.205	2	1	2	1	0	3.5
**HBA**							
NPs	4.756 ± 3.059	4	2	6	4	0	12
ChEMBL	5.599 ± 2.535	5	4	7	3	1	11.5
BindingDB	5.772 ± 2.064	6	4	7	3	2	11.5
**p(X)**							
NPs	-1.330 ± 0.502	-1.495	-1.796	-0.953	0.843	-2	0.108
ChEMBL	-2.234 ± 1.503	-2.252	-3.477	-1.079	2.398	-5.450	1.510
BindingDB	-2.101 ± 1.335	-2.017	-3.049	-1.111	1.939	-5.380	0.959

(MW = molecular weight, LogP = log10(partition coefficient), TPSA = topological polar surface area, HBD = number of hydrogen bond donors, HBA = number of hydrogen bond acceptors, p(X) = $-\log_{10}(X)$)

2 Supplementary Figures

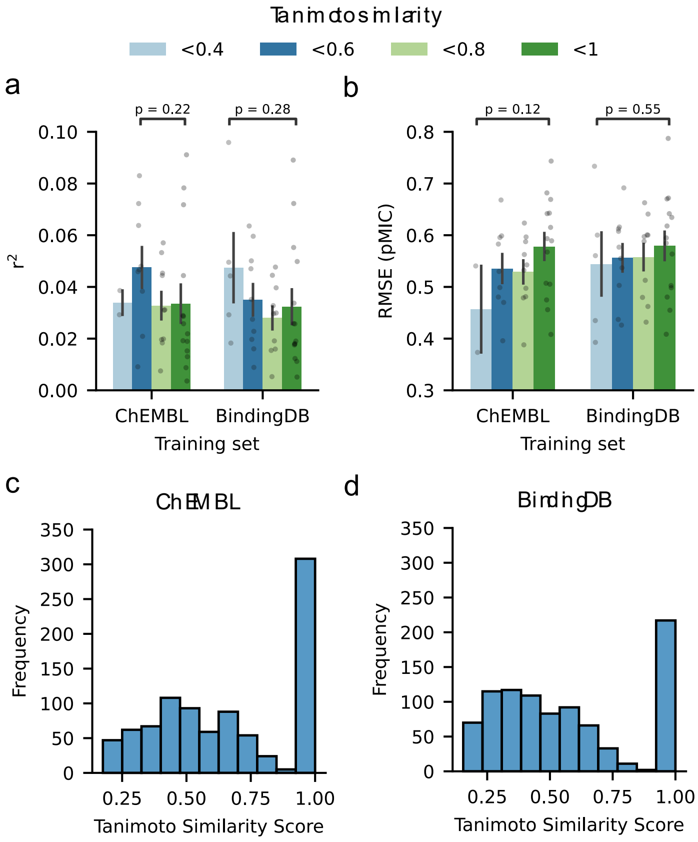

Supplementary Figure 1. **a,b,** Bar plots comparing ActFound's performance on the NPs dataset in terms of (**a**) r^2 and (**b**) RMSE when compounds in the antibacterial NPs dataset with a Tanimoto similarity score <0.4, <0.6, <0.8, and <1 to compounds in the training datasets were used to fine tune the models. Plots show the mean ± s.e.m. performance values across the *n* number of assays used to fine-tune the ChEMBL pre-trained models and the BindingDB pre-trained models. Prior to plotting, the performance values for the assays were averaged across each shot setting used to fine-tune the models. The n value for each dataset is as follows: <0.4 BindingDB $n = 5$, ChEMBL $n = 2$; <0.6 BindingDB $n = 10$, ChEMBL $n = 9$; <0.8 BindingDB $n = 10$, ChEMBL $n = 10$; <1 BindingDB $n = 14$, ChEMBL $n = 14$. Statistical significance between the performance on the entire dataset (Tanimoto similarity < 1) and best comparison dataset were assessed using an independent two-sample *t*-test. **c,d**, Histograms depicting the distribution of Tanimoto similarity scores between compounds in the antibacterial NPs dataset and (**c**) ChEMBL and (**d**) BindingDB.

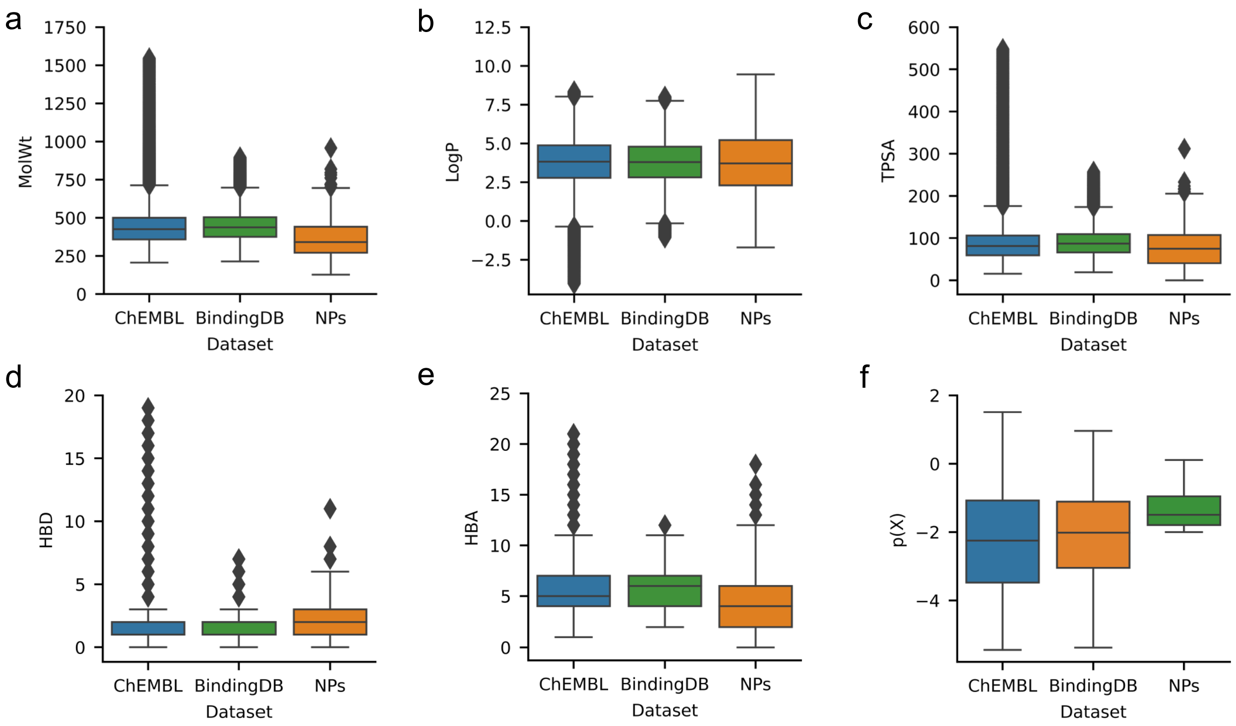

Supplementary Figure 2. Box plots comparing the (**a**) Molecular Weight, (**b**) LogP, (**c**) Topological Polar Surface Area, (**d**) number of Hydrogen Bond Donors, (**e**) number of Hydrogen Bond Acceptors, and (**f**) the negative log of the bioactivity values between the NPs antibacterial fine-tuning dataset and the ChEMBL and BindingDB training datasets. To enable clearer comparisons between datasets, extreme outliers were removed, and the values plotted were restricted to those that fell within the 1st and 99th percentile range. The bounds of the box plots can be found in Supplementary Table 4. An analysis of the entire datasets can be found in Supplementary Table 1.

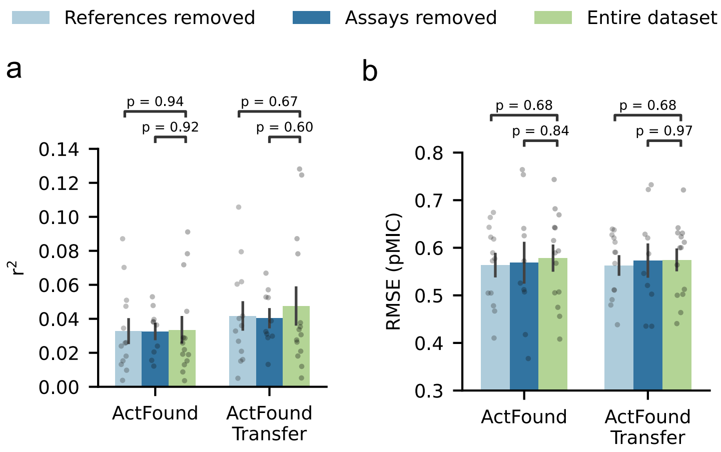

Supplementary Figure 3. Bar plots comparing ActFound and ActFound Transfer's performance on the NPs dataset in terms of (**a**) r^2 and (**b**) RMSE when identical references between the NPs and ChEMBL dataset are removed (*n* = 13), when identical assays between the NPs and ChEMBL dataset are removed (*n* = 10), and when the entire NPs dataset (*n* = 14) was used for fine-tuning. Plots show the mean ± s.e.m. performance values across the *n* number of assays used to fine-tune the ChEMBL pre-trained models. Prior to plotting, the performance values for the assays were averaged across each shot setting used to fine-tune the models. Statistical significance between the performance on the entire dataset and when references and assays were removed were assessed using an independent two-sample *t*-test.

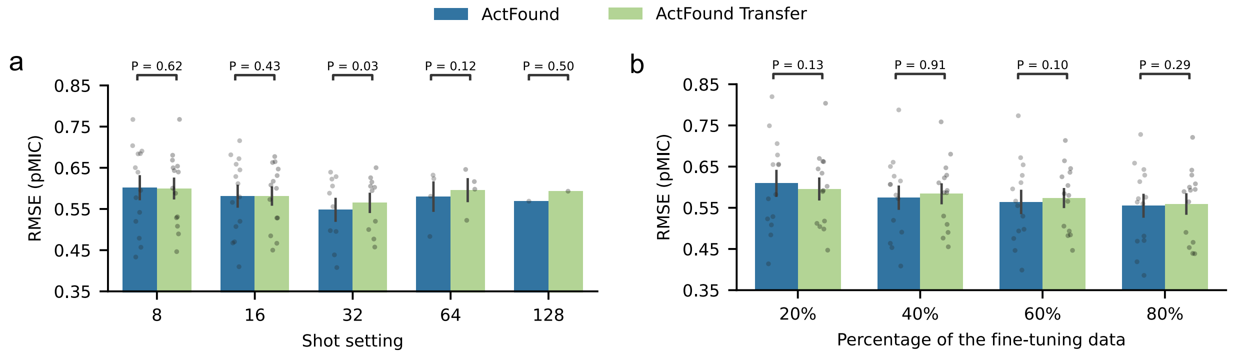

Supplementary Figure 4. Bar plots comparing ActFound and ActFound Transfer's performance in terms of RMSE at the (**a**) 8- (*n* = 14), 16- (*n* = 14), 32- (*n* = 10), 64- (*n* = 4), and 128- (*n* = 1) shot setting and when (**d**) 20 (*n* = 14), 40 (*n* = 14), 60 (*n* = 14), and 80% (*n* = 14) of the assays were used for fine-tuning. Plots show the mean ± s.e.m. performance values across the *n* number of assays used to fine-tune the pre-trained models. Statistical significance between the performance of ActFound and ActFound Transfer at each shot setting was assessed using a one-sided Wilcoxon test.

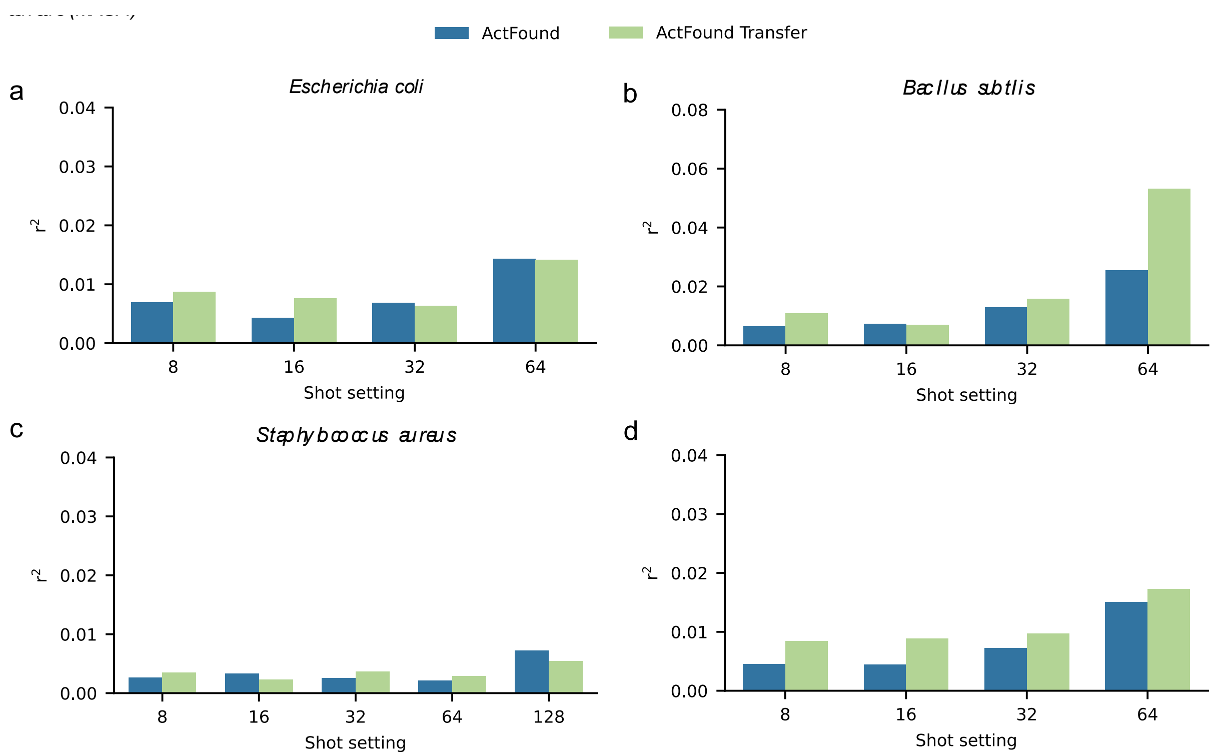

Supplementary Figure 5. Bar plots comparing ActFound and ActFound Transfer's performance in terms of r^2 at the 8-, 16-, 32-, 64-, and 128-shot setting when (**a**) the *E. coli*, (**b**) *B. subtilis*, (**c**) *S. aureus*, and (**d**) *S. aureus* (MRSA) assays were used for fine-tuning. Plots show the mean performance values across the 40 fine-tuning iterations.

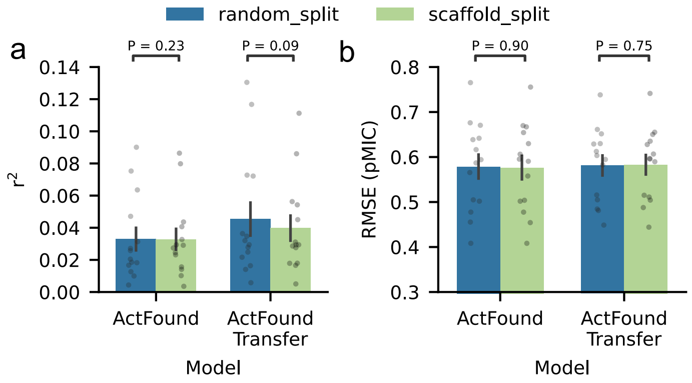

Supplementary Figure 6. Bar plots comparing ActFound and ActFound Transfer's performance on the NPs dataset in terms of (a) r^2 and (b) RMSE when a random split and when a scaffold split was used. Plot shows the mean ± s.e.m. performance values across the $n = 14$ assays used to fine-tune the ChEMBL and BindingDB pre-trained models. Prior to plotting, the performance values for the assays were averaged across each shot setting used to fine-tune the models. Statistical significance between the performance of ActFound on a random split and a scaffold split was assessed using a one-sided Wilcoxon test.

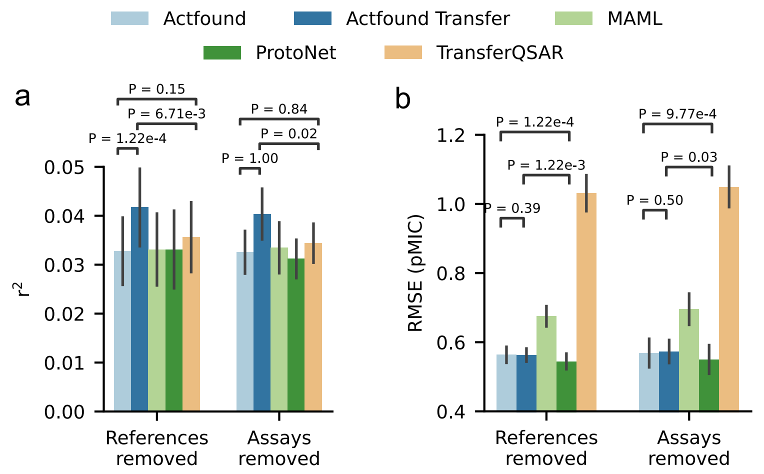

Supplementary Figure 7. Bar plots comparing each model's performance on the NPs dataset in terms of (**a**) r^2 and (**b**) RMSE when identical references between the NPs and ChEMBL dataset are removed (n = 13) and when identical assays between the NPs and ChEMBL dataset are removed (n = 10). Plots show the mean ± s.e.m. performance values across n number of assays used to fine-tune the ChEMBL pre-trained models. Prior to plotting, the performance values for the assays were averaged across each shot setting used to fine-tune the models. Statistical significance between the performance of ActFound, ActFound Transfer, and the best comparison approach were assessed using a one-sided Wilcoxon test.

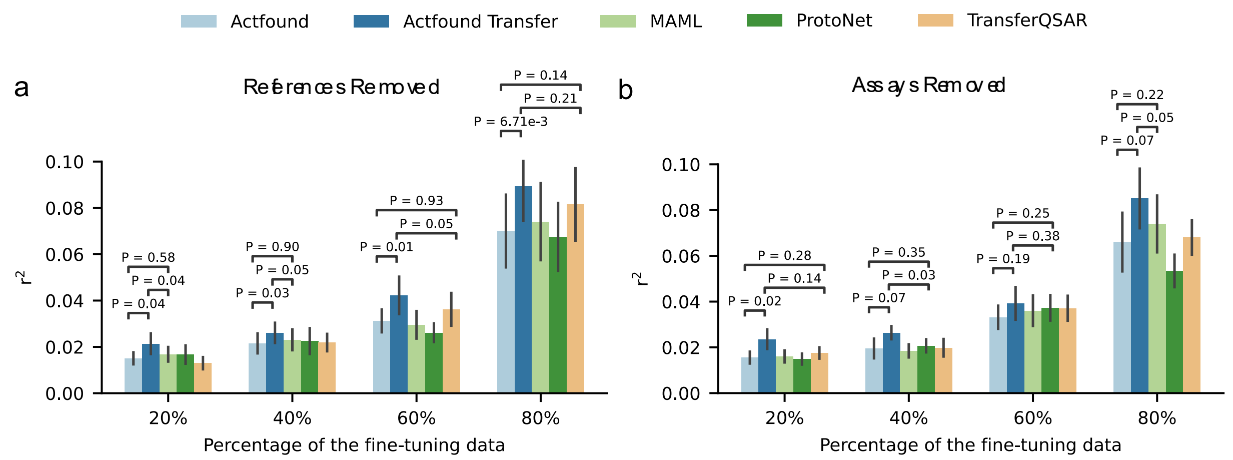

Supplementary Figure 8. Bar plots comparing the models' performances when 20, 40, 60, and 80% of the assays were used for fine-tuning in terms of r^2 (**a**) when identical references between the NPs and ChEMBL dataset are removed (n = 13) and (**b**) when identical assays between the NPs and ChEMBL dataset are removed (n = 10). Plots show the mean ± s.e.m. performance values across n number of assays used to fine-tune the ChEMBL pre-trained models.. Statistical significance between the performance of ActFound, ActFound Transfer, and the best comparison approach were assessed using a one-sided Wilcoxon test.

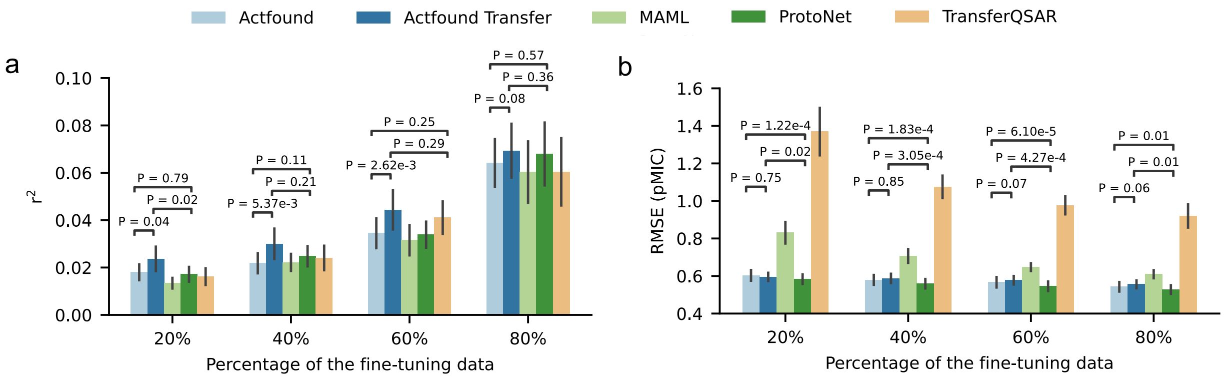

Supplementary Figure 9. Bar plots comparing the models' performances when 20, 40, 60, and 80% of the assays were used for fine-tuning in terms of (**a**) r^2 and (**b**) RMSE when a scaffold split was used. Plots show the mean ± s.e.m. performance values across $n = 14$ assays used to fine-tune the ChEMBL pre-trained models and the BindingDB pre-trained models. Statistical significance between the performance of ActFound, ActFound Transfer, and the best comparison approach were assessed using a one-sided Wilcoxon test.
